# A Multi-Sensor Environmental Perception System for an Automatic Electric Shovel Platform

**DOI:** 10.3390/s21134355

**Published:** 2021-06-25

**Authors:** Xudong Li, Chong Liu, Jingmin Li, Mehdi Baghdadi, Yuanchang Liu

**Affiliations:** 1 School of Mechanical Engineering, Dalian University of Technology, Dalian 116024, China; lixudong2015@mail.dlut.edu.cn; 2 Key Laboratory for Micro/Nano Technology and System of Liaoning Province, School of Mechanical Engineering, Dalian University of Technology, Dalian 116024, China; jingminl@dlut.edu.cn; 3 Key Laboratory for Digital Design and Intelligent Equipment Technology of Liaoning Province, School of Mechanical Engineering, Dalian University of Technology, Dalian 116024, China; 4 Key Laboratory for Precision and Non-traditional Machining Technology of Ministry of Education, School of Mechanical Engineering, Dalian University of Technology, Dalian 116024, China; 5 Department of Mechanical Engineering, University College London, Torrington Place, London WC1E 7JE, UK; m.baghdadi@ucl.ac.uk (M.B.); yuanchang.liu@ucl.ac.uk (Y.L.)

**Keywords:** environmental perception system, electrical shovel, point cloud processing, multiple sensors

## Abstract

Electric shovels have been widely used in heavy industrial applications, such as mineral extraction. However, the performance of the electric shovel is often affected by the complicated working environment and the proficiency of the operator, which will affect safety and efficiency. To improve the extraction performance, it is particularly important to study an intelligent electric shovel with autonomous operation technology. An electric shovel experimental platform for intelligent technology research and testing is proposed in this paper. The core of the designed platform is an intelligent environmental sensing/perception system, in which multiple sensors, such as RTK (real-time kinematic), IMU (inertial measurement unit) and LiDAR (light detection and ranging), have been employed. By appreciating the multi-directional loading characteristics of electric shovels, two 2D-LiDARs have been used and their data are synchronized and fused to construct a 3D point cloud. The synchronization is achieved with the assistance of RTK and IMU, which provide pose information of the shovel. In addition, in order to down-sample the LiDAR point clouds to facilitate more efficient data analysis, a new point cloud data processing algorithm including a bilateral-filtering based noise filter and a grid-based data compression method is proposed. The designed platform, together with its sensing system, was tested in different outdoor environment conditions. Compared with the original LiDAR point cloud, the proposed new environment sensing/perception system not only guarantees the characteristic points and effective edges of the measured objects, but also reduces the amount of processing point cloud data and improves system efficiency. By undertaking a large number of experiments, the overall measurement error of the proposed system is within 50 mm, which is well beyond the requirements of electric shovel application. The environment perception system for the automatic electric shovel platform has great research value and engineering significance for the improvement of the service problem of the electric shovel.

## 1. Introduction

Electric shovels are large and complex engineering machines, which have been widely used in practical applications, such as mining and loading materials in open-pit coal mines [1]. Although the operation of electric shovels is relatively simple, optimal operation productivity is difficult to achieve. For example, during the operation, common issues, including low filling factor, long time-consuming and high safety issues, always exist and can be more serious when electric shovels are handled by inexperienced operators in complex operating environments [2,3]. To address these issues, it is important to equip electric shovels with the capability of automatic operation, so that a unified operation performance can be achieved regardless of operators [4]. 

Therefore, a new automatic electric shovel platform has been designed in this work at Dalian University of Technology (DUT). Such a platform has the features of simple structure, compact overall size and lightweight. To enable an intelligent excavation operation for the designed new platform, an environment sensing/perception system consisting of multiple sensors has been developed and mounted. It provides necessary information that will support the subsequent excavation missions, leading to an intelligent operation electric shovel [5,6].

At present, researchers across the globe have conducted a large number of studies and experiments on environmental sensing/perception systems for different excavating machines [7]. A 1:7 scaling electric shovel prototype, which performs 3D identification of excavating materials by using a single LiDAR, was designed by the Australia Autonomous Systems Laboratory. However, this system could not detect and provide the pose information of the shovel in real-time, making the accuracy of the scanning data largely compromised. A Caterpillar excavator platform using the technology of final echo recognition for environmental detection has been designed. However, there was no relevant sensor to measure the position and attitude of the electric shovel, so it neither could accurately obtain real-time and accurate point cloud data [8,9,10]. Hiroshi Yamamoto from Tsukuba University installed a GPS/INS (inertial navigation system) on an excavator to obtain information such as the direction of the excavator [11]. Color and depth information can be collected by a stereo camera to assist with 3D scene reconstruction. The Missouri University of Science and Technology developed a virtual prototype model of the P&H4800XPC model electric shovel [12] and carried out a simulation analysis. On the small hydraulic press platform, Gu et al., from Northeastern University (China), studied intelligent mining based on vision technology. By installing three cameras on the platform, the current environment image data were obtained, which provided important decision-making information for automatic mining. However, the three-dimensional environmental information obtained through vision would be easily affected by light, rain and snow and cannot be applied to harsh working conditions [13]. Xie et al. used a small hydraulic excavator to build a three-dimensional environment sensing/perception system based on a single-line LiDAR and a camera [14]. These works lack pose information and data fusion.

In 2018, Baidu developed an automated small excavator based on computer vision technology, which scanned the global scene through drone aerial photography and then reconstructed a three-dimensional map of the site. However, it is currently in the experimental stage of a small prototype, which cannot be applied to mine environments with heavy excavation workloads and harsh environments. Aiming at the complex construction environment of mines, Gao G X et al. researched image dust removal processing algorithms based on color histogram, channel compensation, CIELAB color space and other methods and made certain progress. However, these methods are not suitable for processing pictures with large depth changes in the scene and the enhanced image does not conform to the color distribution and the processing effect for severe sand and dust pictures is not ideal [15,16,17,18].

The existing research methods provide basic techniques for the perception of the topography and physiognomy of electric shovels [19,20,21]. However, problems, such as complex structure, low perception accuracy, lack of real-time checking of data processing, still remain, which makes the development of an accurate sensing/perception system for automatic electric shovels essential. Constrained by complex operating environments affected by various weather conditions, a single sensor solution is not capable of fully describing the environment information required for a safe and reliable operation of automatic electric shovels. To overcome these issues, multiple sensors, such as LiDAR, inertial navigation and RTK, can be implemented to work coherently to provide enriched data information [22,23,24,25,26].

This paper endeavors to investigate how to integrate multiple low-cost sensors to provide an accurate 3D sensing/perception of environments and a self-positioning of electric shovels in real-time. To achieve these, a sensing/perception system was designed to provide a 3D point cloud information. Two 2D single-line LiDARs were used in the proposed perception system to scan the nearby topography, which achieved a continuous real-time measurement of the surrounding environment. The system provides the position information of the electric shovel platform and can simplify the structure of the system by utilizing a combined calculation of RTK and GPS/INS.

Over the past decades, researchers have proposed several isotropic algorithms to filter point cloud data. A classic solution is the Laplace smoothing algorithm [27]. Although this method can effectively remove noise, it can blur the sharp features of the model and cause the model to shrink. Based on the Laplace algorithm, Taubin et al. [28] proposed a two-step strategy. After denoising, the model was enlarged to make up for the problem of volume reduction during filtering. Desbrun et al. [29] proposed an isotropic algorithm based on linear smoothing. This work applied linear diffusion and curvature flow to models such as irregular grids to achieve smooth filtering, but some edges were blurred.

Fleishman et al. applied the idea of bilateral filtering algorithm for the image field [30]. The Gaussian variance on the basis of Gaussian filtering was added to the bilateral filtering algorithm, which considered the similarity of the neighborhood pixel values and the Euclidean distance relationship of the neighborhood pixels. The spatial distance of the pixels and the difference between the pixel values determine the weight of the bilateral filtering. On this basis, we combined the bilateral filtering method with the disordered 3D point cloud analysis and transformed the 3D information into the 2D information based on the bilateral filtering calculation through reverse thinking and realized the filtering processing of the point cloud with reasonable parameter conversion. This algorithm can retain edge information while removing edge noise and has good research significance and engineering value to reduce data errors caused by equipment vibrations and non-uniform surface variations [31]. A grid-based data compression algorithm was also proposed to achieve an efficient down-sampling of dense point clouds [32,33]. The Compression processing of point cloud data is necessary to reduce the number of point clouds without affecting the overall accuracy and feature points. This not only saves the system processing memory space, but also improves the efficiency of real-time operations. The hardware and software of the proposed perception system have been tested and validated on the newly built automatic electric shovel platform at DUT. The experimental results demonstrated that the accuracy of the collected 3D point cloud can well meet the expected design requirements. The research on the environmental sensing/sensing system of electric shovel is not only the successful application of multi-sensor fusion sensing technology, but also provides a good theoretical foundation and practical value for the intelligent transformation of the electric shovel.

The remainder of this paper is organized as follows. The related content of Section 2 focuses on the experimental electric shovel platform and the designed perception system. Section 3 focuses on the proposed synchronization algorithm for the collaborative generation of 3D point cloud data from multiple sensors and Section 4 introduces the 3D point cloud data processing method suitable for the work scene of the electric shovel. Section 5 provides a series of experimental results to verify and evaluate the proposed algorithm and Section 6 summarizes the paper and discusses future work.

## 2. The Overall System Construction

**Figure 1:**
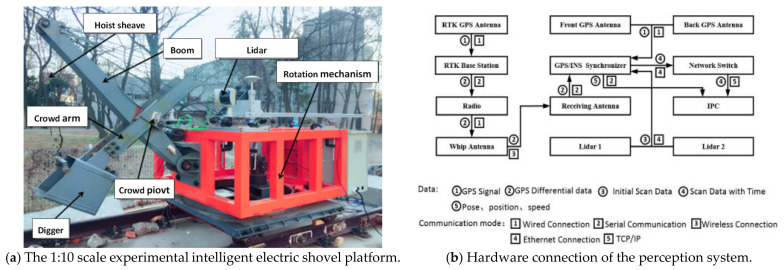
The electric shovel platform and hardware connection.

In order to better undertake the research for electric shovels and comply with the requirements of safety, efficiency and effectiveness, a 1:10 scale experimental platform was designed according to the actual size of electric shovels and the reliability, authenticity and optimality were taken into the account. To ensure safety, the platform was placed on a fixed rail to be constrained. The real machine structure is shown in Figure 1a and it can be observed that the shovel consists of key elements of a practical on-site shovel, such as the boom, the crowd arm, the rotation mechanism and the dipper. During the operation of the electric shovel, the pushing and lifting action can be jointly operated to complete the excavation of the material and, after the excavation is completed, the rotation action is performed to complete the unloading work. Based on the position of the loading vehicle, the proposed electric shovel can also perform the bidirectional rotation.

The main component of the proposed new sensing/perception system for the automatic electric shovel includes two 2D single-line LiDARs, an RTK base station, a mobile station that consists of satellite and INS and a radio station of sending end. To achieve a combined computation of the data obtained from LiDAR and GPS/INS, a hardware synchronizer was designed to synchronize all the data from the sensors. The overall hardware structure of the system is shown in Figure 1b. Moreover, it can be seen that, after the single-line LiDAR and GPS/INS are synchronized by the synchronizer, the data is transmitted to the computer for computation, then a 3D point cloud data of surrounding environment can be generated.

**Figure 2:**
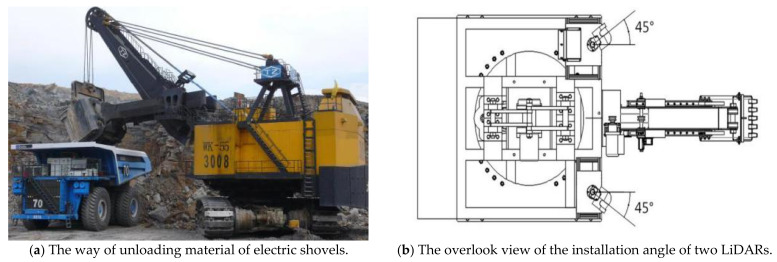

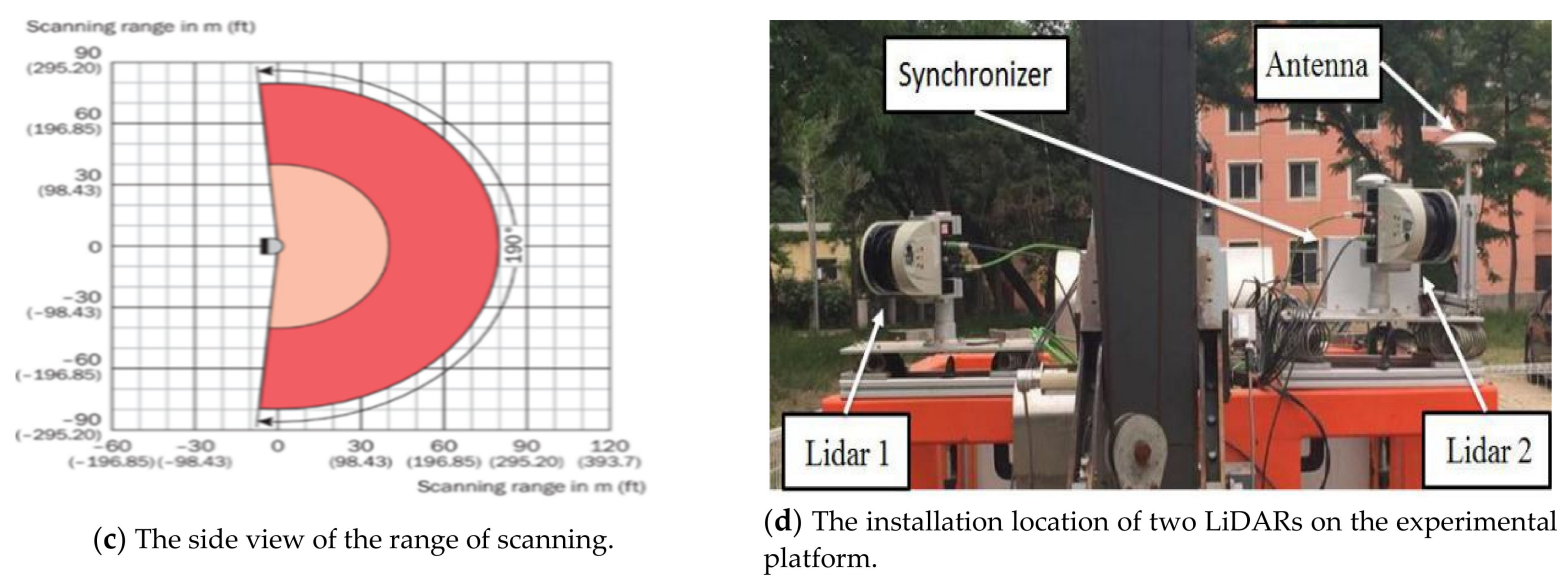
LiDARs installation design principles and physical photos.

As shown in Figure 2a, in practical operating conditions, the electric shovel normally unloads materials from two directions. To achieve a complete scan, two LiDARs were installed at an angle of 45 degrees to the direction of forwarding motion (shown in Figure 2b) with each LiDAR having a scan range of 190 degrees in horizontal plan (shown in Figure 2c).

The final configuration of two LiDARs on the designed electric shovel platform is shown in Figure 2d. In this system, the INS, the LiDAR and the GPS antenna remain apart by a certain distance and form a rigid connection. With regard to how the shovel works, the LiDAR was horizontally installed on the top of the shovel so that the measuring section of LiDAR is perpendicular to the ground. In general, the longer the distance of the baseline of two antennas in the satellite/INS module is, the better the accuracy of the posture measurement. Consequently, the distance between the two antennas should be guaranteed to be over 2 m, when the system is installed. The connecting line of the phase center of the GPS antenna should be accordant or parallel to the advanced direction of the excavator platform. In addition, considering the fact that the actual surface was uneven, a damping spring was installed between the base of the device and the platform to reduce the influence of the instability of the rigid structure.

## 3. The Data Synchronization

**Figure 3:**
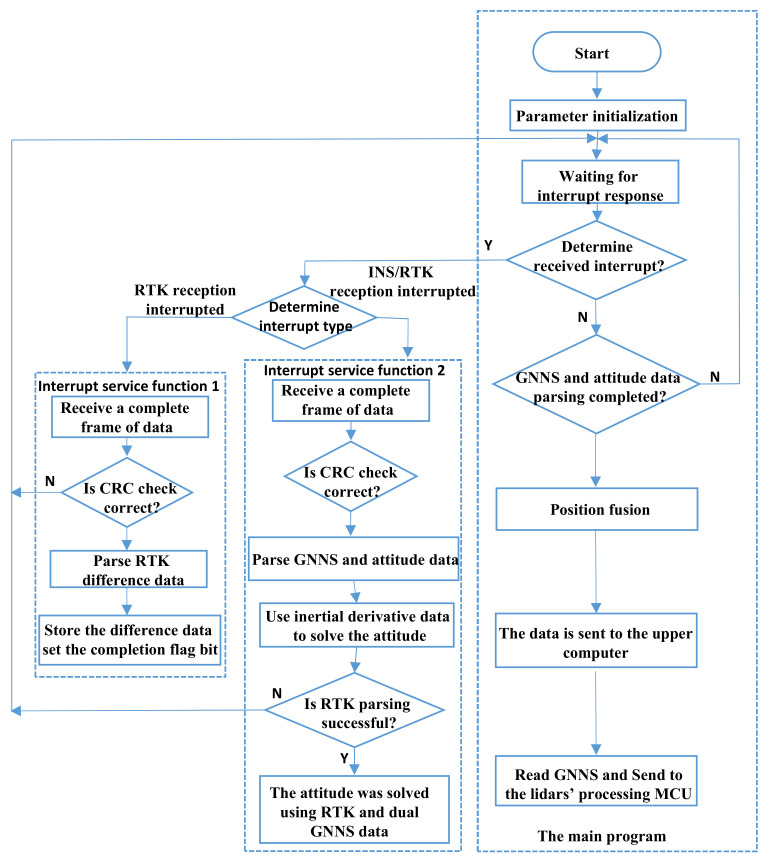
Data synthesis processing MCU program design flow chart.

**Figure 4:**
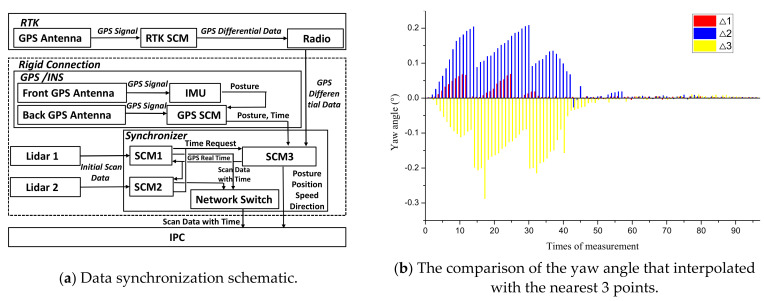

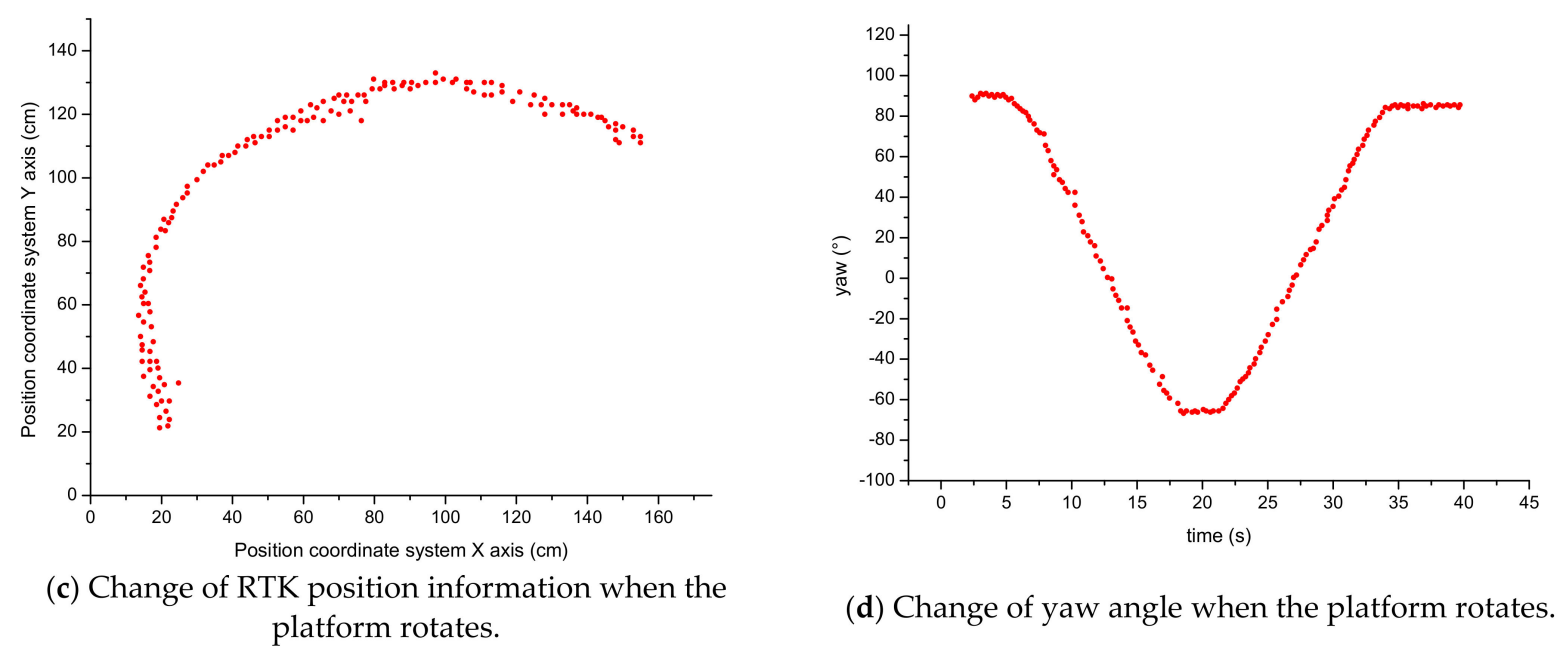
Data synchronization schematic and experimental result diagram.

When the electric shovel starts working, the system first makes a time service to the RTK base station and GPS/INS and, until the time service is successful, the LiDARs can be activated. The flow of data synthesis processing is shown in Figure 3. In the environmental sensing/perception system, the two LiDARs work independently. To splice the data of the two LiDARs, a data synchronization must be carried out using time synchronization. As shown in Figure 4a, all sensor data are synchronized on the basis of the timed GPS/INS time. Whenever there is a frame of LiDAR data transmitted to the SCM (single-chip microcomputer), an interrupt will be triggered and the interrupt program records the time of GPS/INS as the generation time of laser data for this frame. Following such a way, the synchronous SCM time stamps each frame data scanned by the two LiDARs and communicates with the computer through the UDP protocol mode. The RTK base station provides a fixed reference coordinate for the mobile station after it is timed and sends the differential data, which have the time stamp to the GPS/INS for further computation, so that a more accurate position and posture can be obtained.

The data acquired by a 2D LiDAR need to be combined with the RTK pose information before generating 3D point cloud data. In the proposed system, since the working frequencies of LiDAR (50 Hz) and RTK (100 Hz) are different, if the pose information provided by the RTK is directly used for conversion, a large error will be formed. In this paper, the Lagrange interpolation method is used to estimate the pose information of the LiDAR data by referring to the RTK data. Since the 2D LIDAR data need to be converted into 3D data in real-time, the interpolation method needs to find the closest data from the 2D point cloud to the RTK data stream acquired in real-time. Then, according to the RTK data interpolation result, the pose information of LiDAR can be determined.

Matching the successful laser time *T*, the position information corresponding to the adjacent *n* groups of RTK time is subjected to Lagrange interpolation operation and used as the position information for LiDAR point cloud. Assuming RTK data are of the form of (*x*, *y*, *z*) and the RTK data in the *x*-axis corresponding to time *t_i_* are *x_i_*, the Lagrange interpolation polynomial can be expressed as:

(1) $$L_{nx}(T)=\sum_{i=0}^{n-1}x_{i}H_{i}(T)$$

where

(2) $$H_{i}(T)=\prod_{i}\in D_{k}\frac{T-t_{i}}{t_{k}-t_{i}},(D_{k}=\{i\left|i\neq k,i\in \{0,1,\ldots n-1\}\}\right.)$$

When *n* = 3, the above formula can be simplified to the following equation:

(3) $$L_{3x}(T)=\frac{\left(T-t_{1})(T-t_{2}\right)}{\left(t_{0}-t_{1})(t_{0}-t_{2}\right)}x_{0}+\frac{\left(T-t_{0})(T-t_{2}\right)}{\left(t_{1}-t_{0})(t_{0}-t_{2}\right)}x_{1}+\frac{\left(T-t_{0})(T-t_{1}\right)}{\left(t_{2}-t_{0})(t_{2}-t_{1}\right)}x_{2}$$

According to the same principle, we can calculate the data of the *y*-axis and the *z*-axis using the RTK Lagrange interpolation *L_3y_* (*T*) and *L_3z_* (*T*) at time T as:

(4) $$L_{3y}(T)=\frac{\left(T-t_{1})(T-t_{2}\right)}{\left(t_{0}-t_{1})(t_{0}-t_{2}\right)}y_{0}+\frac{\left(T-t_{0})(T-t_{2}\right)}{\left(t_{1}-t_{0})(t_{0}-t_{2}\right)}y_{1}+\frac{\left(T-t_{0})(T-t_{1}\right)}{\left(t_{2}-t_{0})(t_{2}-t_{1}\right)}y_{2}$$

(5) $$L_{3z}(T)=\frac{\left(T-t_{1})(T-t_{2}\right)}{\left(t_{0}-t_{1})(t_{0}-t_{2}\right)}z_{0}+\frac{\left(T-t_{0})(T-t_{2}\right)}{\left(t_{1}-t_{0})(t_{0}-t_{2}\right)}z_{1}+\frac{\left(T-t_{0})(T-t_{1}\right)}{\left(t_{2}-t_{0})(t_{2}-t_{1}\right)}z_{2}$$

In order to achieve the above functions, we have designed a comprehensive data processing program. Its main workflow is as follows:

(1) Parameter initialization. When the embedded system is powered on and reset, the entire system will initialize and run to realize the IO port mode setting and the initialization of some functions and parameters.
(2) Wait for the interrupt trigger and determine the interrupt type to execute the corresponding interrupt service function.
(3) RTK data reception and analysis interrupt program processing: receive, judge and analyze the standard differential data sent by the RTK base station through the serial port interrupt function and use it to correct the GNSS data.
(4) INS/RTK data analysis interrupt program processing: receive data sent by INS/RTK through the serial port interrupt function, where the priority setting of the receiving interrupt function is lower than the priority of RTK differential data reception. According to the differential data sent by the analytical RTK, the INS/RTK outputs the GNSS message information after compensation calculation, including information such as meter head, time, yaw angle and pitch angle. According to the message information, the attitude information and time data are extracted and the acceleration, angular velocity, magnetometer and quaternion data of the received inertial navigation are filtered and fused.
(5) Time query request interrupt program processing: according to the interrupt request response of the time query, the GNSS information is time-calculated.
(6) Posture data transmission processing: after the posture data are calculated, the posture data calculated by the inertial navigation and the position and posture information calculated by the dual GNSS antennas are sent to the host computer through the serial port.

Only by performing real-time pose detection on LiDAR, the point cloud data can be accurately calculated, which is one of the key points to multi-sensor fusion. Because the LiDAR poses information is provided dependent upon the interpolated RTK data at any time, the accuracy of the interpolation is important. As the main aim of this experiment is to validate whether the proposed interpolation algorithm is capable of approximating the yaw angle to the true value as accurately as possible, two sets of experiments have been undertaken: (1) the platform was first configured to be static and true yaw angle was 0°; (2) the platform was rotating with a constant angular velocity. For both experiments, after obtaining the RTK data, the multi-interpolation was performed and the results were compared with the actual yaw angle to verify the accuracy.

Figure 4b shows the comparison between the interpolation result of GPS/INS yaw angle and the three nearest RTK-INS yaw angles at the time *T* (Δ*1*, Δ*2*, Δ*3*), when the platform is static. It can be seen that the interpolation result is very consistent with the adjacent time, i.e., when the system has just started, the error caused by the synchronizer delay is about 0.2°. When the system tends to run smoothly, the error drops and the interpolation result can be regarded as the true value of the laser yaw angle.

The experiment results obtained from the rotating platform are shown in Figure 4c,d. The change of RTK position information during platform rotating is shown in Figure 4c, which shows that the plane motion trajectory of LiDAR/INS is generally smooth. The small jump in some positions is caused by the fluctuation of the original position data of RTK within a certain range, which will not affect the final point cloud precision. Figure 4d shows that the change of yaw angle is relatively stable when the platform moves back and forth. When the platform is stationary (ab, cd, ef), the yaw angle remains unchanged. During the rotation process (bc, de), the yaw angle always maintains a good linear relationship with the LiDAR time.

## 4. Point Cloud Data Processing

A cost-effective 3D point cloud generation method has been proposed in this paper. By employing two single-line LiDARs, 2D point cloud data obtained via a single scan can be converted into a 3D point cloud by making the platform tilt within a certain range. To obtain complete 3D environment information around the shovel, two single-line LiDARs installations have overlapping perspectives. In the data processing, corresponding point cloud registration is also required to reconstruct the complete 3D data based on the environmental characteristics of the 2D point cloud data.

The management, filtering and feature extraction of point cloud data are important in the comprehensive processing of 3D point cloud data. The processing of point cloud data requires the support of mature point cloud processing algorithms and related function libraries. However, albeit the simple mechanism of the 3D point cloud generation process, issues of limited data accuracy and high data redundancy remain unsolved. To rectify these issues, two new methods are employed. First, after obtaining the preprocessed data, a point cloud filtering algorithm is used to remove noise and outliers to reduce the impact of noise on point cloud registration and feature point extraction. Second, because the single-line LiDAR used in this system can detect depths of up to 80 m with high accuracy, a substantial amount of data can be generated within a single scan period, which can influence point cloud feature extraction and real-time calculation processing. On the basis of maintaining the shape characteristics of the point cloud, the amount of data should be compressed and reduced.

### 4.1. Noise Filtering Based on Bilateral Filtering

When acquiring point cloud data, a series of noise points will appear due to reasons such as sensors’ accuracy, platform vibration, non-uniform surface variation and electromagnetic wave interference. In particular, when using the proposed system to undertake the 3D environment scanning process, in addition to the noise points generated by the random errors in the above-mentioned measurement, there are some outliers that deviate from the main point due to obstacles. In the point cloud process, the filtering process is the first step, which has a great influence on the subsequent point cloud registration, feature extraction, surface reconstruction and visualization process [34].

We need to limit the filtering point according to the actual application conditions and use the filtering algorithm to modify the attributes of the point, to sample the data. Bilateral filtering, which is a non-linear spatial filtering method, can preserve the edge information of an image while filtering out digital image noise in the field of image processing. The bilaterally filtered neighborhood pixel weight is the product of the spatial proximity factor and the pixel similarity factor. Among them, the spatial proximity factor decreases with the increase of the Euclidean distance between the neighbor point and the midpoint and the pixel similarity factor decreases with the increase in the pixel value difference between them [35].

The filtering of the point cloud data acquired by the 3D LiDAR is similar to the filtering of the digital image. The point clouds are 3D data describing the surface properties of an entity, whereas images are two-dimensional data. Therefore, we improved a 3D to 2D inverse conversion method to achieve bilateral filtering of 3D point clouds. When using bilateral filtering to process point cloud data, neighborhood data should be used to establish a plane approximation to represent the surface. At this time, the distance between each neighbor point to the plane can be regarded as the pixel point intensity difference in the bilateral filtering and the distance between each neighbor point relative to the central scanning point can be regarded as the pixel point distance in the bilateral filtering.

**Figure 5:**
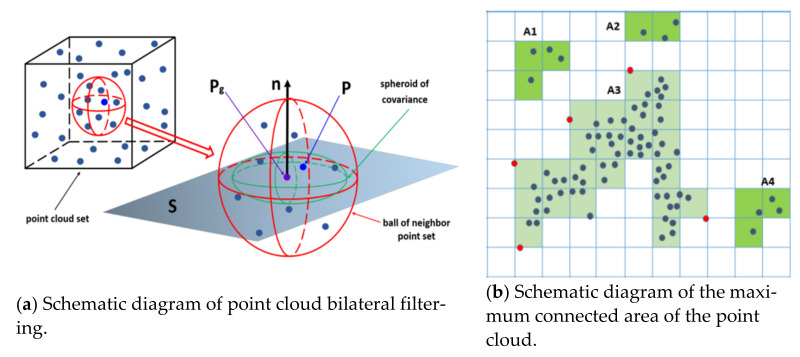
Schematic diagram of two filtering methods.

As shown in Figure 5a, it is necessary to establish the k-neighborhood K(*p*) of the sample point *p*, to calculate the fit plane of the neighborhood point *p* and to use the fit plane as the view plane *S*. The view plane *S* is the primary datum for evaluating subsequent data magnitudes and is used to provide important information, such as the projection direction. *p’* is the projection point of p on the *S* plane. Finally, we need to calculate the spatial proximity (*v*) of *p’* and the neighborhood points on the *S* plane. The height (*h*) of point *p* over the tangent plane *S* is synonymous with the pixel values of an image.

The relationship between the initial point cloud data and the filtered point cloud data is defined as:

(6) $${p_{i}}^{\prime }=p_{i}+\alpha \cdot n$$

where *p_i_’* indicates the filtered point cloud, *p_i_* (*i* = 1, 2, 3…*n*) indicates the initial point cloud and *n* is the unit normal vector of the fitting plane *S*(*p*) over the neighborhood point. The center of gravity of the *p_i_* point cloud set is *p_g_*, which can be calculated as:

(7) $$p_{g}=\frac{1}{n}\sum p_{i}$$

The covariance matrix of the *p_i_* point cloud set is *C*, with the feature value of *C* being *λ_k_* and the feature vector of *C* being V_k_, which are calculated as:

(8) $$C={\left[\begin{array}{c} p_{i}-p_{g}\\ \ldots \\ p_{i}-p_{g} \end{array}\right]}^{T}{\left[\begin{array}{c} p_{i}-p_{g}\\ \ldots \\ p_{i}-p_{g} \end{array}\right]}^{},i=1,2,3\ldots n$$

(9) $$C\times V_{k}=\lambda_{k}\times V_{k}$$

*α* (as shown in Equation (6)) indicates bilateral filter factor as:

(10) $$\alpha =\frac{\sum p_{j}\in N(p_{i})W_{c}(\left\|p_{i}-p_{j}\right\|)W_{s}(\left\|(n_{i},n_{j})-1\right\|)(p_{i}-p_{j},n_{i})}{\sum p_{j}\in N(p_{i})W_{c}(\left\|p_{i}-p_{j}\right\|)W_{s}(\left\|(n_{i},n_{j})-1\right\|)}$$

where *W_c_* is the Gaussian kernel function of the bilateral filter function in the spatial domain, which is used to adjust the smoothness of the bilateral filtering and *W_s_* is a Gaussian kernel function in the frequency domain, which is used to control the degree of feature retention. *W_c_* and *W_s_* can be calculated as:

(11) $$W_{c}(x)=e^{\frac{-x^{2}}{2{\sigma_{c}}^{2}}}$$

(12) $$W_{s}(y)=e^{\frac{-y^{2}}{2{\sigma_{s}}^{2}}}$$

where *x* = || *p_i_ − p_j_* || indicates the Euclidean distance from the sampling point to *p_i_* and the point *p_j_* in the neighborhood. The key steps of the algorithm are as follows:

1. Initializing point cloud data, for each data point *p_i_* in the region, searching for a point in its K(*p_i_*);
2. Calculate *W_c_*(*x*) and *W_s_*(*y*) according to Equations (11) and (12), then substituting the results into Equation (10) and calculating *α*;
3. Substituting *α* and normal vector *n* into Equation (6) to obtain new data points;
4. Calculate all data points, output point cloud data after denoising and the algorithm ends.

For the filtering of point cloud data, we also use a combination of removing outliers and Gaussian filtering. Outliers that are far from the main body of the point cloud need to be removed by filtering. Firstly, point cloud data are divided into cells and the cell size is adjusted to determine the most suitable connected area as the main body of the point cloud. The data in the point cloud grid that are far away from the main body are removed to achieve the deletion of outliers. The maximum connected area algorithm is to treat the divided point cloud cells as pixels in a two-dimensional image and connect adjacent cells that contain point cloud data together. The maximum connected area is the main body of the point cloud. Other cells that are not connected into a large area are outliers that need to be eliminated, as shown in Figure 5b. Among them, A1, A2 and A4 are outlier point cloud cells and A3 is the largest connected area of the point cloud and it is also the main body of the point cloud.

After removing the global outliers, there are still some outliers in the local part of the point cloud body. Based on the point cloud kd-tree data structure, the Gaussian method can be used to filter the point cloud k neighborhood. Firstly, calculate the average distance from a point to each point in the k neighborhood. These distances normally follow a normal distribution and those that do not meet the normal distribution can be considered as local outliers.

### 4.2. Grid-Based Data Compression

The point cloud data collected by the LiDAR are relatively dense, which requires a proper data compression method to make the generated data compliant with the data storage capacity of the onboard computer. In this paper, a voxelization method is used to achieve data down-sampling and the method can reduce the amount of data of the point cloud, while largely maintaining the shape features of the point cloud. This algorithm divides the points into multiple cubes, calculates the average point of the coordinates in each cube and replaces all the points in the cube with the average point to complete the point cloud compression. The key feature of the algorithm lies in the determination of the size of the cube. The sampling step is dependent upon the side length of the cube as the interval length. In general, the setting of the cube side length needs to be larger than the neighboring points of the point cloud to achieve an optimized compression effect [36].

**Figure 6:**
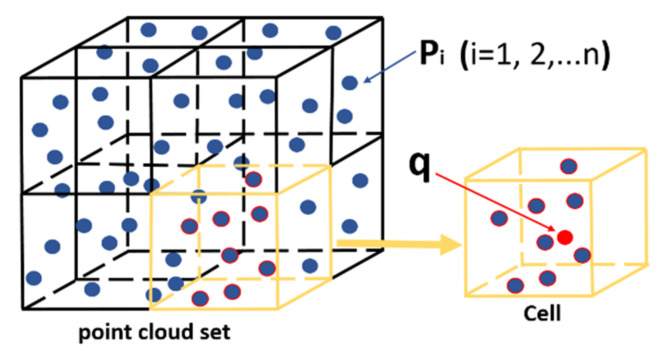
Schematic diagram of grid-based data compression.

As shown in Figure 6, *p_i_* represents the point in space, *i* = 1, 2, …, n, where n represents the total number of point cloud data. *Cell_[i][j][k]_* represents a well-divided grid, *I* ∈ {1, 2, …, *CellNum_x_*}, j ∈ {1,2,…,*CellNum_y_*}, k ∈ {1, 2, …, *CellNum_z_*}. *CellNum_x_*, *CellNum_y_* and *CellNum_z_* are the number of grids divided on the *X*, *Y* and *Z* axes. The minimum and maximum values of *P_i_* on the *X*, *Y* and *Z* axes are *x_min_*, *y_min_*, *z_min_*:

(13) $${CellNum}_{x}=\frac{x_{\max}-x_{\min}}{l}$$

(14) $${CellNum}_{y}=\frac{y_{\max}-y_{\min}}{l}$$

(15) $${CellNum}_{z}=\frac{z_{\max}-z_{\min}}{l}$$

There are m points in a divided grid, which can be represented by the point *q*:

(16) $$q_{x}=\frac{\sum_{1}^{m}x_{i}}{m}$$

(17) $$q_{y}=\frac{\sum_{1}^{m}y_{i}}{m}$$

(18) $$q_{z}=\frac{\sum_{1}^{m}z_{i}}{m}$$

For a given point cloud model, its structure is generally not a simple cube. Before the division, the minimum bounding body of the point cloud model needs to be constructed to divide the minimum bounding volume. Through the specific analysis of the point cloud model, the steps of the spatial partitioning method are as follows:

1. The extremum values in the *X*, *Y* and *Z* dimensions are recorded separately when the point cloud data are read;
2. Construct the minimum bounding volume and set the split resolution to *M*;
3. The minimum bounding body is divided into objects and it is determined whether the number of points in the bounding body is smaller than the resolution *M* and if so, the dividing ends; otherwise, the minimum bounding body is divided;
4. Obtain a series of point cloud unit blocks;
5. Calculate the average point of the coordinates in each cube, then replace all points in the cube with the average point to complete the compression of the point cloud.

The vertices in each unit block are relatively close and contain a moderate number of points. In the subsequent processing, we no longer use the entire point cloud model as the operation object, but the unit blocks as the object, greatly improving the efficiency of the algorithm.

Although the processing of point cloud filtering and down-sampling is a standard method, the combination of processing methods and the personalized setting of parameters can achieve a more significant performance in engineering based on ensuring accuracy and efficiency, which is useful for the development of engineering equipment and provide theoretical and practical basis.

## 5. Experimental Results and Analysis

To verify the effectiveness of the designed sensing/perception system for the electric shovel platform, scenes with multiple types of objects are selected. The experiment was arranged in a dedicated open space on the campus of DUT. This experimental site has the characteristics of a large scene range, a wide field of view and a stable GNSS signal reception, which can meet the needs of large-scale detection of LiDAR, and the experimental site has landmark objects, such as buildings, trees, fences, etc., which can facilitate achieving the error analysis of the scan results and the actual size of the objects.

In order to verify the measurement accuracy of the environmental awareness system under various environments, the experiment was scheduled from July to October (temperature of 20°–40°) and from January to April (temperature of −20°–20°). The LiDAR image, which is the main measurement experimental equipment with an angle, ranges from 0° to 190° with a minimum angular resolution of 0.2°. The detection range of the LiDAR is 0–80 m and it has multiple echo detection functions, which can effectively eliminate the effects of smog, light rain and the refraction of transparent objects.

Through actual measurement of LiDAR and processing of the system, the raw point cloud information of the site was first obtained and displayed. Then, by undertaking the procedures of denoising, filtering and compression, the final point cloud dataset can be obtained. To identify the accuracy of the proposed system, several representative objects were selected, such as the fence and the door objects, for dimensional measurement and the results were compared with the actual measurement data.

**Figure 7:**
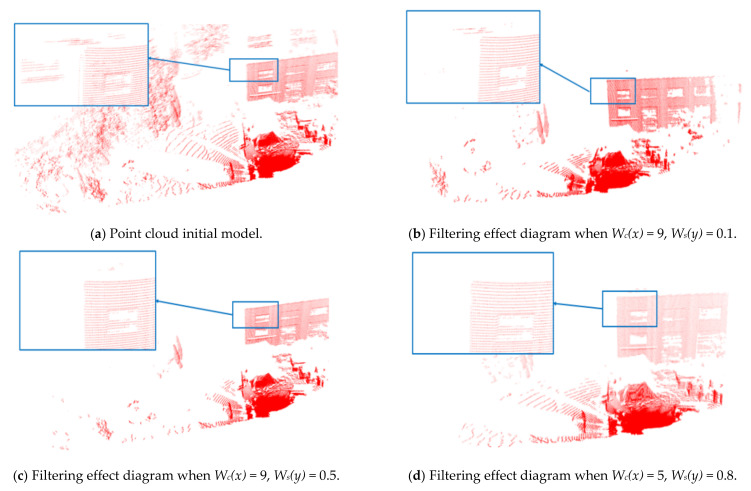

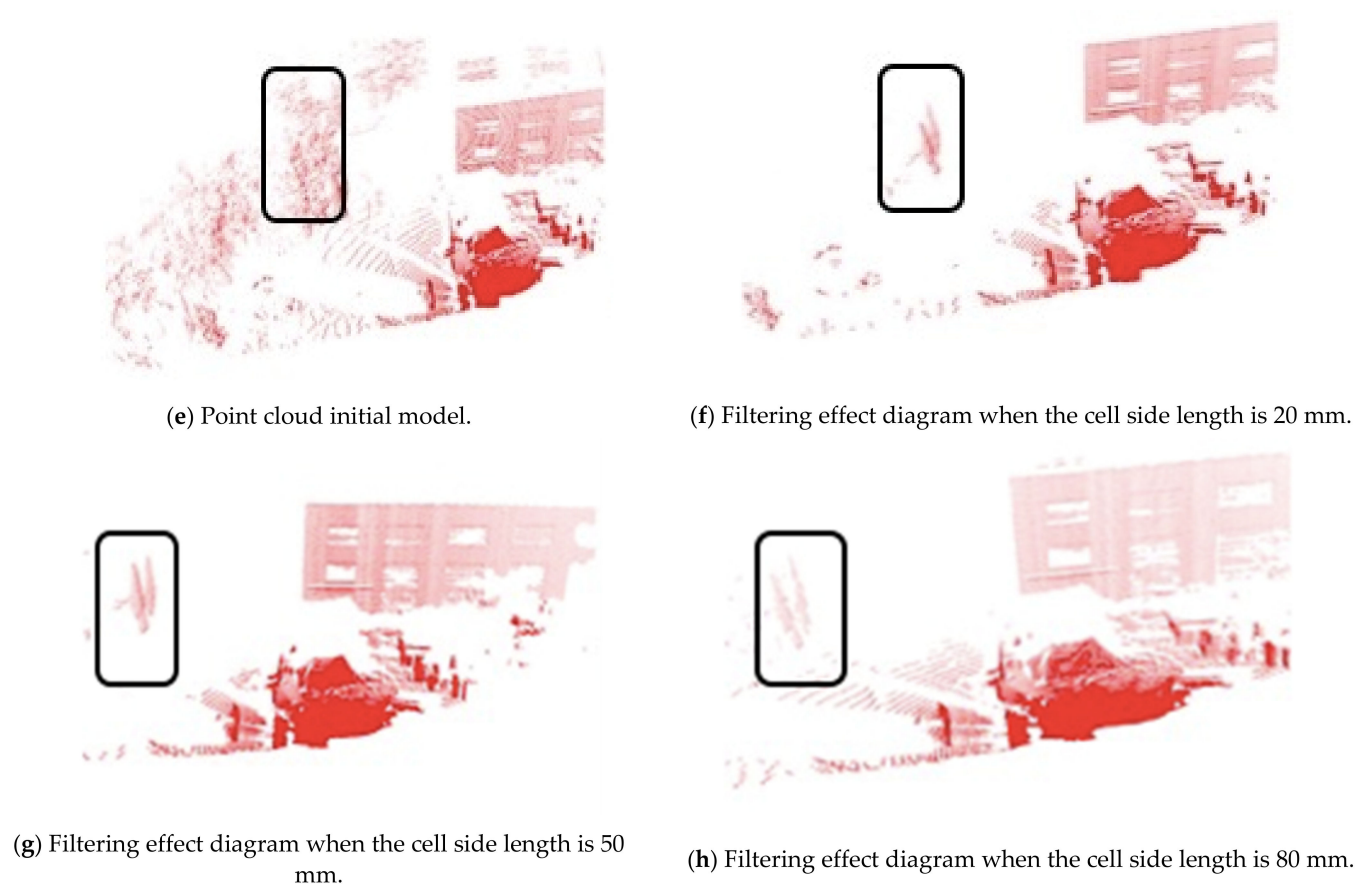
Bilateral filtering, removing outliers and Gaussian denoising effect of point cloud model under different parameters.

Noise reduction experiments of bilateral filtering, removing outliers and Gaussian filtering are performed on a point cloud model. Comparing the experimental results by setting different parameters, the point cloud processing effect diagram is shown in Figure 7.

**Figure 8:**
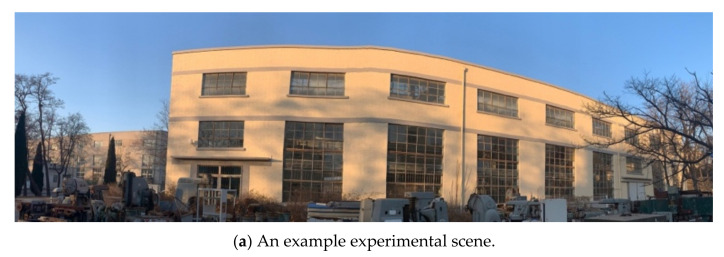

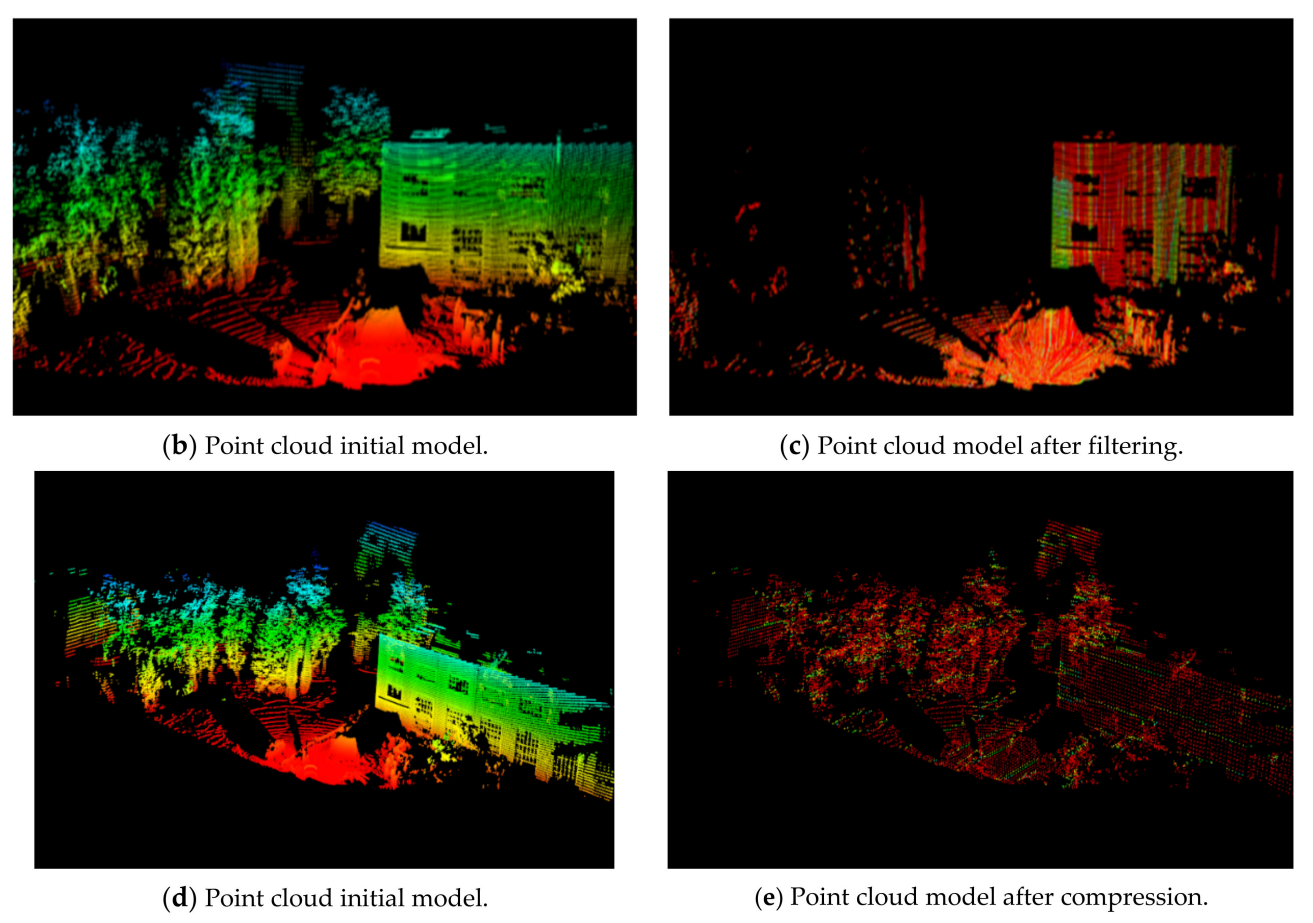
Comparison of point cloud with and without filtering and compression.

It can be seen from the bilateral filtering denoising results that the salient features and detailed features of the model are preserved. Increasing *W_s_*(*y*), the feature retention is better, but the smoothness is worse; increasing *W_c_*(*x*), the smoothness is enhanced, but the detail features are lost. It also can be seen that the combination of the method of removing outliers and Gaussian filtering will play a certain filtering role. However, as the size of the grid increases, the feature points of the point cloud cannot be guaranteed, such as trees and windows of the building. At the same time, the point cloud at the edge of the measured object has a certain effect and the volume has also changed. Therefore, compared with Gaussian filtering and removing outliers, bilateral filtering has a good effect of enhancing the edge of the image and feature points. The filtering effect is shown in Figure 8a–c.

**Figure 9:**
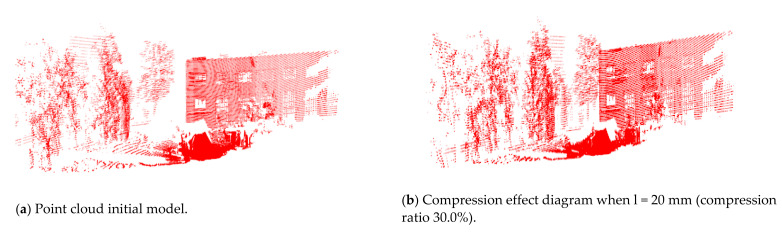

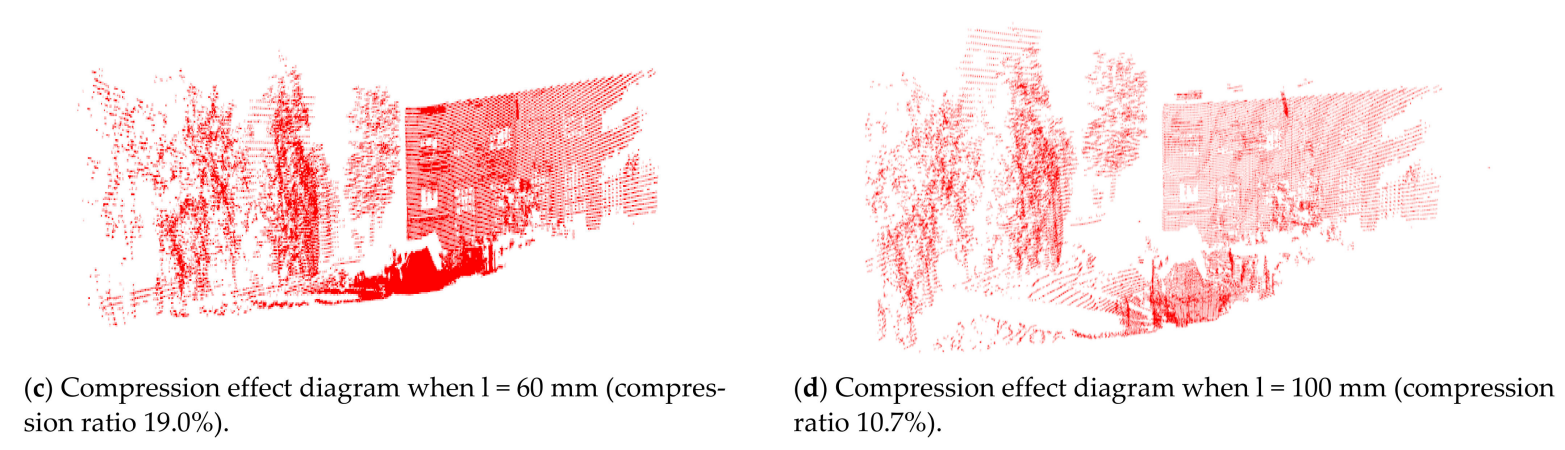
Compression effect of point cloud model under different side lengths.

The results of the compression process depend on the different side lengths (l), so we compare the results of compression for different side lengths (l). The compression effect is shown in Figure 8a,d,e. It can be seen, from Table 1, that side lengths have a great influence on the compression ratio. The higher the length is, the greater the point cloud is compressed to have less point cloud data. It should also be noted that the compression process does not alter the scene feature greatly. It can be seen from Figure 9 that when the side length is equal to 100 mm, the point cloud diagram can still accurately reflect the characteristics and size of the actual environment.

For different point cloud models, the minimum boundary subject of the point cloud model needs to be constructed to autonomously divide the minimum boundary volume. Then use the measurement error value specified by the actual operation of the electric shovel to inversely infer the maximum boundary volume. By setting the safety threshold, the system autonomously compares the relationship between the two volumes and selects the best segmentation value under the premise of ensuring safety accuracy. Therefore, considering the electric shovel has the characteristics of single target work and fixed maximum error value in actual work, we independently divide it through data comparison and optimal selection. In our study, not only it has a certain accuracy and reliability, but a more intelligent development of the electric shovel also provides a feasible solution to the practical engineering problem.

**Figure 10:**
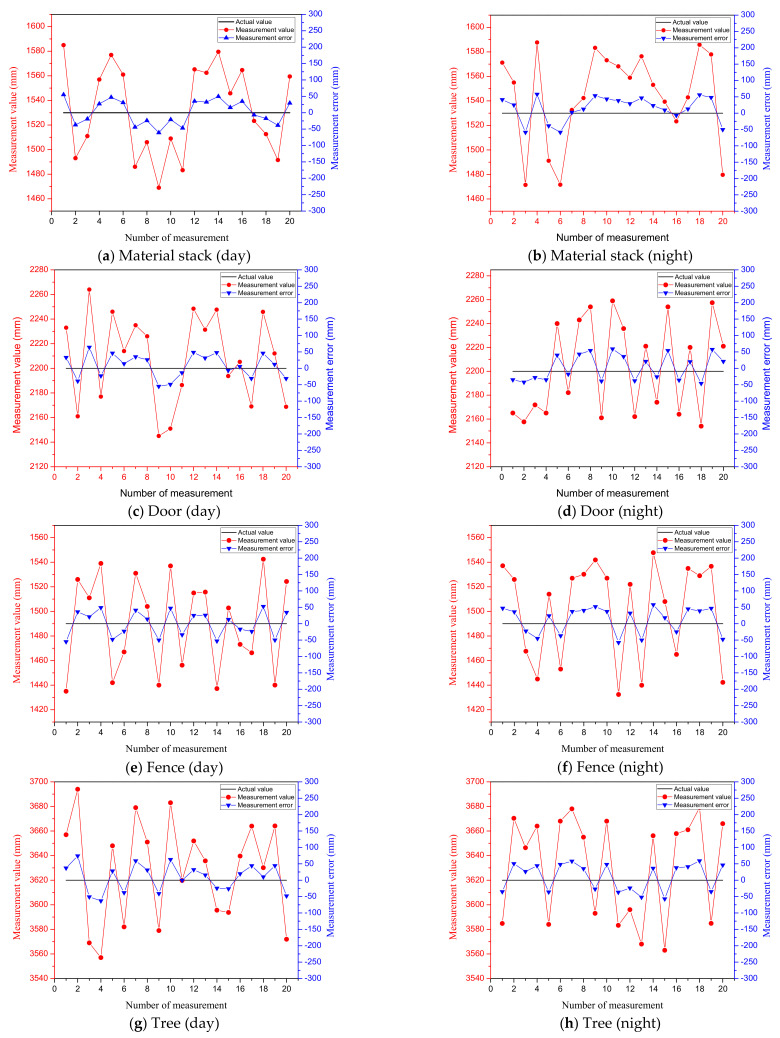

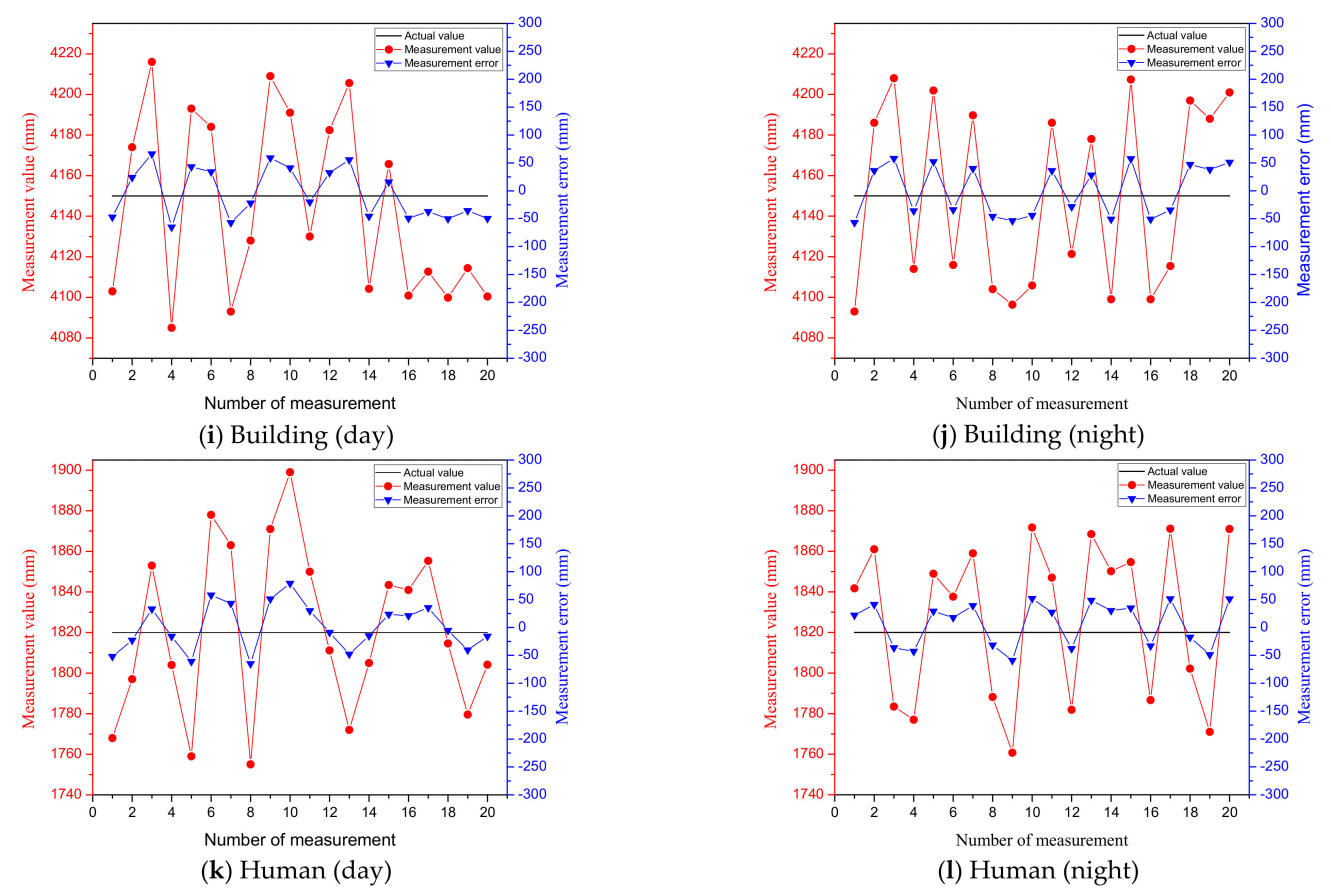
Actual value, measurement value and measurement error chart when measuring different objects.

**Figure 11:**
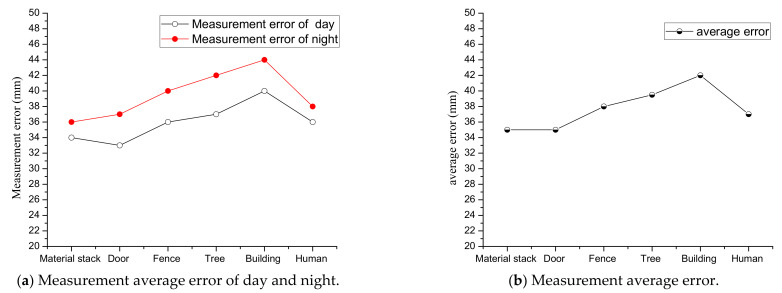
Measurement average error.

To further demonstrate the effectiveness of the algorithm, we selected six representative objects at the test site to analyze the measurement results. Each object is within a linear distance of 5–20 m from the LiDAR and each test object is subjected to multiple LiDAR scanning measurements to ensure the accuracy of the results. For LiDAR, due to the influence of multiple factors, such as laser ranging sensor, scanning angle sensor, data processing, etc., the measurement data are also uncertain. According to the equipment parameters and theoretical calculations, the measurement error of the LiDAR selected in this paper is within 40 mm (within the range of 100 m). To analyze the overall performance of the measurement system, the objects are scanned during both day and night and the error is analyzed with the actual size of the objects measured by a ruler. The actual sizes of the objects are shown in Table 1, the reference size values provided in Table 1 are manually obtained using measurement tools, so they have a certain measurement uncertainty. The measurement error of the tools we use is within 10 mm. The measured values and errors of different objects day and night are shown in Figure 10. Among them, the red points indicate the measured values and the blue points indicate the measurement errors. It is shown that measurements for all objects during day and night are relatively accurate, where the measurement errors can be retained within small values for all cases. A summarized result is shown in Figure 11, where the red points in (a) indicate the average error of scanning six object sizes at night, the black points indicate the average error of scanning six object sizes during the day and (b) indicates the overall average error obtained after the day and night data are summarized. It can be seen that the errors of scanning objects at night are slightly larger than that of daytime, but the overall measurement error of the system is within 50 mm, which is accurate enough to ensure the performance of the electric shovel.

**Figure 12:**
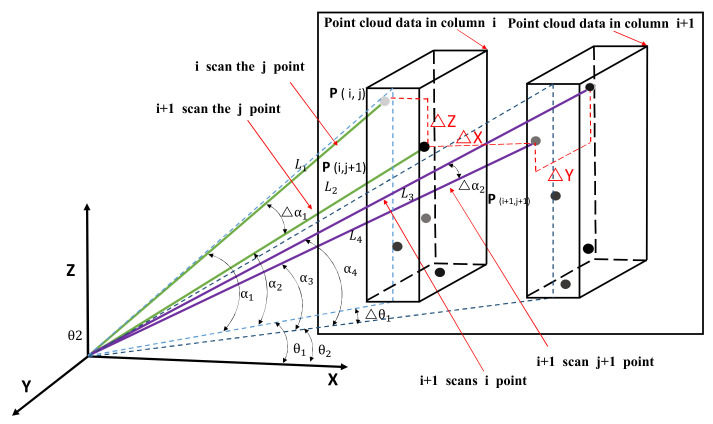
Schematic diagram of 2D single-line LiDAR scanning process.

The detection accuracy of a 3D environmental scanning system refers to the minimum distance that the system can detect two points in space along the *X*, *Y* and *Z* axes. The process of 2D LiDAR scanning two columns of point clouds continuously is shown in Figure 12. It can be seen from the figure that ∆*X*, ∆*Y*, ∆*Z* can measure the detection accuracy of the LiDAR along the *X*, *Y*, *Z* axis. ∆*X* is the minimum detectable distance along the horizontal *X* axis in two adjacent columns of point clouds, ∆*Y* is the minimum detectable distance along the depth *Y* axis of the same column of point clouds and ∆*Z* is the minimum detectable distance along the vertical *Z* axis.

(19) $$\left\{\begin{array}{c} \Delta X=L_{3}\cos\alpha_{3}\cos\theta_{2}-L_{2}\cos\alpha_{2}\cos\theta_{1}\\ \Delta Y=L_{3}\cos\alpha_{3}\sin\theta_{2}-L_{4}\cos\alpha_{4}\sin\theta_{2}\\ \Delta Z=L_{1}\sin\alpha_{1}-L_{2}\sin\alpha_{2} \end{array}\right.$$

According to the formula, the accuracy of the 3D scanning system on the *X* axis is related to the detection distance *L*, the LiDAR launch angle $\alpha_{1}$ and $\alpha_{2}$ and the electric shovel rotation angle $\theta_{1}$ and $\theta_{2}$. The detection distance L can be directly measured by the LiDAR and the difference Δ*α* between $\alpha_{2}$ and $\alpha_{3}$ is equal to the angular resolution of the LiDAR at 0.5°. The difference Δ*θ* between $\theta_{1}$ and $\theta_{2}$ represents the angular error of the yaw angle, which is related to the scanning frequency *f_L_* of the LiDAR, the time matching error Δ*T* of the LiDAR and the yaw angle and the angular speed *ω* of the device working rotation. The specific relationship is shown in equation 20:

(20) $$\Delta \theta =\left(\frac{1}{f_{L}}+\Delta T\right)\omega$$

$\theta_{1}$, $\theta_{2}$ can be obtained through the attitude module and $\alpha_{2}$ and $\alpha_{3}$ can be obtained from LiDAR data messages, but the acquisition process is more difficult. Generally, the error analysis of ∆*Y* and ∆*X* is omitted and the accuracy error in the ∆*Z* direction is mainly analyzed. Due to the characteristics of the 2D LiDAR line scanning, the accuracy error in the *Z*-axis direction is often analyzed, as shown in the formula ∆*Z* in the equation (19).

Based on the above error analysis, the error between the accuracy of the 3D point cloud and the theoretical measurement accuracy after the fusion of the 3D scanning system of the electric shovel is compared. Through data analysis, we draw the following conclusions: at different detection distances, the accuracy of the fused 3D point cloud data meets the theoretical accuracy, the error value is negligible relative to the size of the equipment itself and the working environment range and the measurement accuracy of the scanning system can meet the requirements of the electric shovel.

## 6. Conclusions and Future Works

In this paper, a novel and efficient sensing/perception system was proposed and designed for an automatic electric shovel platform. A 1:10 scaled shovel experimental platform was designed to provide an efficient and accurate tested for the sensing/perception system. To meet the work requirements of accurately acquiring 3D environmental information, based on the study of the working method and working environment of the electric shovel, a comparative analysis of the widely used environmental perception method and a single-line LiDAR with the advantages of large measurement range, high precision and low cost was adopted as the ranging unit of the system. Then, through the combination of RTK and IMU, accurate pose information of the system could be obtained and a centimeter-level precision could be achieved. With the purpose of being capable of performing real-time scanning of full angles, based on the structure (easy to produce blind spots) and loading characteristics of the electric shovel (loading on both sides), a global 3D point cloud could be generated by the combination of two single-line LiDARs with reasonable layouts and the rotary motion of the platform. Through the precise timing of GPS, the data collected by two LiDARs were time-stamped for synchronization. Comparing the principles and experimental results of point cloud processing methods, the bilateral filtering method was used to denoise the collected point cloud to reduce data errors caused by equipment vibrations and non-uniform surface variations. Finally, the grid method was used to compress the 3D point cloud without sacrificing system accuracy, achieving high operational efficiency. Based on considering the practicality of the electric shovel project, a large number of comprehensive experiments (day and night, light rain) were performed. From the results, we can see that the overall measurement performance of the system is stable and the overall error is within 50 mm. The research on the environmental sensing/perception system of the electric shovel is not only a successful application of multi-sensor fusion perception technology, but also provides a good theoretical foundation and practical value for the intelligent transformation of the electric shovel.

Although through design and research, the experimental results of the environmental sensing/sensing system of the electric shovel can meet the technical requirements of the operation. However, on the basis of ensuring the long-term stability of the system, we also need to continuously improve the system and integrate technology. The feature extraction of point cloud information is a key factor to obtain accurate global 3D environmental information, which is directly related to the identification of targets in the mining scene and the decision-making of intelligent mining. In future work, it is planned to quickly and accurately extract point cloud features, global point cloud splicing and multi-target recognition in the construction area. On the basis of ensuring construction safety, improve the accuracy and reliability of the environmental perception system and carry out the next step of excavation trajectory planning and operation control research to provide the basis for the overall intelligent operation of the platform.

## Figures and Tables

**Table 1:** Comparison of measurement size and actual size of marked point cloud compressed.

Measurement Target	Material Stack	Door	Fence	Tree	Building	Human
Actual value of height (mm)	1530	2200	1490	3620	4150	1820

## Data Availability

Not Applicable.

## References

[1] William H., Mark K. (2013). Open Pit Mine Planning and Design.

[2] Awuah-Offei K., Frimpong S. (2011). Efficient cable shovel excavation in surface mines. Geotech. Geol. Eng..

[3] Orlemann E. (2003). Power Shovels: The World’s Mightiest Mining and Construction Excavators.

[4] Hendricks C., Scoble M.J., Peck J. (1989). Performance monitoring of electric mining shovels. Trans. Inst. Min. Met. Sect. A.

[5] Patnayak S., Tannant D.D. (2005). Performance monitoring of electric cable shovels. Int. J. Surf. Min..

[6] Guilherme J.M., David C.R., Surya P.N.S. (2014). Iterative Autonomous Excavation.

[7] Dunbabin M., Corke P. (2006). Autonomous Excavation Using a Rope Shovel. J. Field Robot..

[8] Anthony S., John B. (1999). A Robotic Excavator for Autonomous Truck Loading. Auton. Robot..

[9] Singh S., Cannon H. Multi-Resolution Planning for Earthmoving. Proceedings of the International Conference on Robotics and Automation.

[10] Rowe P.S. (1999). Adaptive Motion Planning for Autonomous Mass Excavation.

[11] Hiroshi Y., Masaharu M. Development of the Autonomous Hydraulic Excavator Prototype Using 3-D Information for Motion Planning and Control. Proceedings of the 2010 IEEE.SICE International Symposium on System integration, (Oral session).

[12] Wardeh M., Frimpong S. (2016). Virtual Prototyping and Simulation of P&H Electric Rope Shovel-4800 XPC.

[13] Gu Y.M. (2010). Research on Image Processing Technology in Intelligent Excavator Vision System.

[14] Xie K. (2016). Development of Intelligent Operation Test System for Excavator Based on Environmental Recognition Platform.

[15] Gao G., Lai H., Jia Z., Liu Y., Wang Y. (2020). Sand-dust Image Restoration Based on Reversing the Blue Channel Prior. IEEE Photonics J..

[16] Shi Z., Feng Y., Zhao M., Zhang E., He L. (2019). Let You See in Sand Dust Weather: A Method Based on Halo-Reduced Dark Channel Prior Dehazing for Sand-Dust Image Enhancement. IEEE Access.

[17] Cheng Y., Jia Z., Lai H., Yang J., Kasabov N.K. (2020). A Fast Sand-Dust Image Enhancement Algorithm by Blue Channel Compensation and Guided Image Filtering. IEEE Access.

[18] Wang J., Pang Y., He Y., Liu C. (2016). Enhancement for Dust-Sand Storm Images. International Conference on Multimedia Modeling.

[19] Kwame A., Samuel F. (2007). Cable shovel digging optimization for energy efficiency. Mech. Mach. Theory.

[20] Thomas P. (2010). Navigation Signal Processing for GNSS Software Receivers.

[21] Ryde J., Hillier N. (2009). Performance of laser and radar ranging devices in adverse environmental conditions. J. Field Robot..

[22] Yoo H., Kim Y. (2017). Development of a 3D local terrain modeling system of intelligent excavation robot. KSCE J. Civil. Eng..

[23] Zhou Y., Li Q., Chu L., Ma Y., Zhang J. (2020). A measurement system based on internal cooperation of cameras in binocular vision. Meas. Sci. Technol..

[24] Li R.B., Liu J.Y., Zhang L. (2014). LIDAR/MEMS IMU integrated navigation (SLAM) method for a small UAV in indoor environments. Inert. Sens. Syst..

[25] Atia M.M., Liu S., Nematallah H. (2015). Integrated indoor navigation system for ground vehicles with automatic 3-D alignment and position initialization. IEEE Trans. Veh. Technol..

[26] Chang L., Niu X., Liu T. (2019). GNSS/INS/LiDAR-SLAM Integrated Navigation System Based on Graph Optimization. Remote Sens..

[27] Vollmer J., Mencl R., Müller H. (1999). Improved Laplacian smoothing of noisy furface meshes. Comput. Graph. Forum.

[28] Taubin G. A signal processing approach to fair surface design. Proceedings of the 22nd Annual Conference on Computer Graphics and Interactive Techniques.

[29] Desbrun M., Meyer M. Implicit fairing of irregular mesher using diffusion and curvature flow. Proceedings of the 26th Annual Conference on Computer Graphics and Interactive Techniques.

[30] Fleishman S. (2005). Robust moving least-squares fitting with sharp features. ACM Trans. Graph..

[31] Fleishman S., Drori I., Cohen-Or D. (2003). Bilateral mesh denoising. ACM Trans. Graph..

[32] Chen Z.G., Zhang T.Y., Cao J., Zhang Y.J., Wang C. (2018). Point cloud resampling using centroidal Voronoi tessellation methods. Comput.-Aided Des..

[33] Liu B.L., Zhang F.M., Qu X.H., Shi X.J. (2016). A Rapid Coordinate Transformation Method Applied in Industrial Robot Calibration Based on Characteristic Line Coincidence. Sensors.

[34] Li W.L., Xie H., Zhang G., Li Q.D., Yin Z.P. (2016). Adaptive Bilateral Smoothing for a Point-Sampled Blade Surface. IEEE/ASME Trans. Mechatron..

[35] Yan J.F., Deng K.Z., Xing Z.Q. (2013). 3D Laser Scanning Point Cloud Filtering Based on Least Squares Fitting. Bull. Surv. Mapp..

[36] Han X.F., Jin S., Wang M.J. (2017). A review of algorithms for filtering the 3D point cloud Signal Processing. Image Commun..

